# Intermittent Hypoxia Training for Treating Mild Cognitive Impairment: A Pilot Study

**DOI:** 10.1177/1533317519896725

**Published:** 2020-01-06

**Authors:** Hong Wang, Xiangrong Shi, Hannah Schenck, James R. Hall, Sarah E. Ross, Geoffrey P. Kline, Shande Chen, Robert T. Mallet, Peijie Chen

**Affiliations:** 1 Departments of Pharmacology and Neuroscience, University of North Texas Health Science Center, Fort Worth, TX, USA; 2 Shanghai University of Medicine & Health Sciences, Shanghai, China; 3 Institute for Healthy Aging, University of North Texas Health Science Center, Fort Worth, TX, USA; 4 Department of Internal Medicine, University of North Texas Health Science Center, Fort Worth, TX, USA; 5 Departments of Physiology and Anatomy, University of North Texas Health Science Center, Fort Worth, TX, USA; 6 Shanghai University of Sport, Shanghai, China

**Keywords:** cerebral tissue O_2_ saturation, cerebrovascular response, cognitive function, elderly, hypoxemia

## Abstract

Although intermittent hypoxia training (IHT) has proven effective against various clinical disorders, its impact on mild cognitive impairment (MCI) is unknown. This pilot study examined IHT’s safety and therapeutic efficacy in elderly patients with amnestic MCI (aMCI). Seven patients with aMCI (age 69 ± 3 years) alternately breathed 10% O_2_ and room-air, each 5 minutes, for 8 cycles/session, 3 sessions/wk for 8 weeks. The patients’ resting arterial pressures fell by 5 to 7 mm Hg (*P* < .05) and cerebral tissue oxygenation increased (*P* < .05) following IHT. Intermittent hypoxia training enhanced hypoxemia-induced cerebral vasodilation (*P* < .05) and improved mini-mental state examination and digit span scores from 25.7 ± 0.4 to 27.7 ± 0.6 (*P* = .038) and from 24.7 ± 1.2 to 26.1 ± 1.3 (*P* = .047), respectively. California verbal learning test score tended to increase (*P* = .102), but trail making test-B and controlled oral word association test scores were unchanged. Adaptation to moderate IHT may enhance cerebral oxygenation and hypoxia-induced cerebrovasodilation while improving short-term memory and attention in elderly patients with aMCI.

## Introduction

Intermittent hypoxia (IH) can inflict harm or confer benefit, depending on the frequency, duration, and intensity of the hypoxia exposures.^1,2^ Brief bouts of high-intensity IH applied up to 8 to12 h/day^3-5^ to model obstructive sleep apnea (OSA) in rats, increase arterial pressures,^3-5^ intensify inflammation,^6^ impair neurobehavioral function,^7,8^ and worsen ischemic injury of heart^9^ and brain.^10^ In contrast, mild to moderate intensity IH training (IHT) programs (eg, 9%-12% O_2_, 30-90 min/day for 3-5 weeks) afford robust protection of myocardium^11-13^ and brain^14-18^ in animals. The IH intensity and cumulative hypoxia duration per session appear to be critical determinants of beneficial versus detrimental responses to IH.^2^

Increasingly, training with low-dose hypoxic exposures is being applied as nonpharmacological therapy for various pathological disorders in humans. Burtscher et al^19^ reported that normobaric IHT (alternating 3-5 minutes of exposure to 10%-14% O_2_ and 3-minute normoxic recovery, 3-5 cycles per daily session, 15 sessions over 3 weeks) increased peak aerobic capacity of older men with or without coronary artery disease. Haider et al^20^ documented that a similar IHT regimen improved baroreflex sensitivity and autonomic cardiovascular function in patients with chronic obstructive pulmonary disease. Intermittent hypoxia training also has been applied to lower arterial pressures in patients with hypertension,^21^ improve quality of life and physical performance in patients with heart failure^22^ and spinal cord lesions,^23^ and decrease serum glucose concentrations and enhance glucose tolerance in prediabetic patients.^24^ Recently, Bayer et al^25^ reported that a hypoxia–hyperoxia regimen alternating 4- to 7-minute exposures to 10% to 14% O_2_ and 2- to 4-minute exposures to 30% to 40% O_2_ for 4 to 8 cycles/session, 3 sessions/week for 5 to 7 weeks, combined with physiotherapy, occupational therapy, and cycling, augmented cognitive performance on the Dementia-Detection and Sunderland Clock-Drawing tests as well as physical performance on the 6-minute walk test in geriatric patients. However, IHT’s impact on cognitive performance in patients with amnestic mild cognitive impairment (aMCI) has not been reported.

Intermittent hypoxia exposures inevitably lower arterial oxygen saturation (SaO_2_)^26^ and, in turn, cerebral tissue O_2_ saturation (ScO_2_) while enhancing cerebral perfusion or cerebrovasodilation.^27^ Nonetheless, most previous IH studies did not document the participants’ ScO_2_ and SaO_2_. Establishing the relationships between IH, SaO_2,_ and ScO_2_ may facilitate clinical application of IH and foster understanding of the mechanisms of therapeutic adaptation. It remained to be determined whether elderly adults with cognitive impairments would benefit from IHT programs readily tolerated by healthy young adults.^26,27^ This pilot clinical study addressed 2 objectives: first, to demonstrate that elderly patients with aMCI could tolerate cyclic, brief, moderate hypoxemia, sufficient to lower SaO_2_ to approximately 70% within 5 minutes; and second, to examine whether IHT could improve cognitive function and physiological responses to hypoxia in elderly patients with aMCI. Cognitive performance, along with cardioventilatory and cerebrovascular function, arterial pressures, SaO_2,_ and ScO_2_ were assessed in elderly adults with aMCI and compared before and after an 8-week IHT program.

## Methods

### Participants

All participants provided a written consent which was approved by the University of North Texas Health Science Center’s Institutional Review Board for Protection of Human Subjects. Fourteen nonsmokers with aMCI were recruited. The assessment and/or diagnosis of MCI was based on Petersen et al’s criteria^28^ including the patient’s and/or his/her family members’ complaints of memory problem, cognitive decline exceeding that considered to represent normal aging, but intact independent functioning, and no diagnosis of Alzheimer’s disease (AD) and/or dementia, and, in addition, a mini-mental status examination (MMSE) score 1 standard deviation below the group mean of the participant’s age and education cohort, and/or an absolute MMSE score between 18 and 26. The patients were evaluated by licensed practitioners in the Memory Disorders Clinic of Geriatric Assessment and Planning Program at the University of North Texas Health Science Center. The inclusion criteria included that the presence of aMCI and the patient’s ability and willingness to visit the laboratory and tolerate intermittent-hypoxia ventilation via an air-cushioned, disposable face-mask. Patients were included who had controlled stabilized chronic conditions ≥1 year duration, including hypertension, coronary artery disease, ischemic stroke, diabetes or metabolic disease, chronic bronchitis, degenerative arthritis, and/or other aging-related chronic conditions. Exclusion criteria were MMSE score <18; diagnosis of dementia, moderate to severe cognitive impairment or AD; current enrollment in another clinical trial or interventional study; current smoker; uncontrolled chronic conditions including hypertension (systolic/diastolic pressures >140/90 mm Hg with medications), diabetes, chronic renal failure, nephritis, lung fibrosis, emphysema, infectious disease, cancer, or congenital heart disease; recurrent acute conditions such as chest pain, stroke, migraine, headaches, seizures or epileptic episodes, or exacerbation of asthma or allergic rhinitis, myocardial ischemia or infarct, a finding of second or third degree atrioventricular block on electrocardiogram; had or was expecting a major surgery or transplant; or neurological disorders including Parkinson’s disease, amyotrophic lateral sclerosis, vertigo, Tourette’s syndrome, Huntington’s disease, schizophrenia, or bipolar disorder.

Prior to enrollment, all participants visited the laboratory for orientation and assessment of their cardiovascular, respiratory, and tissue oxygen responses to hypoxia. During the orientation, participants were familiarized with the laboratory, study procedures, equipment, and measurements; and their IHT tolerance was determined. Five patients were excluded from the study because of ScO_2_ below 50% while breathing room air (one patient), phobic reaction to the facemask (2 patients), and multiple premature ventricular contractions or arrhythmia during hypoxia breathing (2 patients). Another 2 participants began but did not complete the IHT program because of unanticipated schedule conflicts and a nonstudy related surgery, respectively. Seven participants diagnosed with aMCI completed the 8-week IHT program. Table 1 summarizes the characteristics of these 7 patients, 6 of whom had histories of hypertension managed with medications.

**Table 1.** Characteristics of Study Participants.

Patients (n = 7) With MCI	1 Man, 6 Women
Age	69.3 ± 2.7 years (range 58-78 years)
Body mass index	28 ± 4 kg/m^2^ (5 overweight or obese)
Patients with treated hypertension (n = 6)	On one or more medications: Ca^2+^ channel blockers, angiotensin-converting enzyme inhibitors, angiotensin II receptor antagonists, β-adrenoceptor blockers, diuretics
Other conditions	3 with combined hypercholesterolemia and hypertension 1 with combined type-II diabetes and hypertension 2 hypothyroid, 1 with hypertension
Education	14.5 ± 1.0 years (range 12-20 years)

Abbreviation: MCI, mild cognitive impairment.

### Procedures

After completing the orientation, all eligible patients underwent baseline evaluation of cognitive function, followed on a separate day by assessment of baseline physiological function. The patients then completed an IHT program consisting of 8 cycles per IH sessions of alternately breathing 10% O_2_ and room air, each for 5 minutes, 3 sessions per week for 8 weeks. Physiological function was re-evaluated 1 to 2 days after the last IH session, and post-training cognitive function was assessed 1 to 2 days after the physiological reassessment. All participants were instructed to maintain their regular routine and medications throughout the study.

### Measurements

#### Cognitive assessment

Cognitive function was assessed by a licensed geriatric neuropsychologist using MMSE, California verbal learning test-second edition (CVLT-II short version), digit span (forward and backward), trail making test-B (TMT-B), and controlled oral word association test (COWAT) before and after the 8-week IHT program. All tests were conducted between 10:00 and 11:30 Am, with the patient in the seated position.

#### Physiological assessment

Cardiovascular, respiratory, and tissue O_2_ variables at rest and during 5-minute hypoxia exposures were measured for physiological assessment 1 to 2 days before starting and 1 to 2 days after completing the IHT program. During the test, the patient’s heart rate (HR) was monitored beat-to-beat by a standard electrocardiography limb lead. Continuous systolic arterial pressures (SAP) and diastolic arterial pressures (DAP) were monitored noninvasively by double finger cuffs placed on the proximal phalanges of the left index and middle fingers (CNAP 500, Graz, Austria). Mean arterial pressure (MAP) was estimated as (SAP + 2·DAP)/3. Systemic arterial O_2_ saturation (SaO_2_) was continuously monitored from the right earlobe by a radiometer sensor (TOSCA 500, Radiometer America Inc, Westlake, Ohio). The probe was maintained at 42°C to dilate and thereby arterialize the subcutaneous capillary blood in the earlobe. Regional ScO_2_ of the prefrontal cortex was monitored by near infrared spectroscopy (Somanetics, 5100 INVOS Cerebral Oximeter, Troy, Michigan) with a sensor placed on the right side of the forehead. Preliminary studies confirmed that ScO_2_ on right and left sides shows the same pattern and magnitude in response to hypoxic exposures in elderly patients with MCI. Middle cerebral artery flow velocity (V_MCA_) was monitored by transcranial Doppler sonography using a 2 MHz probe (EZ-Dop DWL System, Singen, Germany) placed on the left temporal window. Cerebral vascular conductance (CVC) was estimated as mean V_MCA_/MAP. These measurements and the techniques of transcranial Doppler sonography combined with near infrared spectroscopy have been applied in our previous studies.^27,29-32^

Continuous inspired and expired fractions of O_2_ and CO_2_ were measured using a mass spectrometer (Perkin-Elmer, 1100 Medical Gas Analyzer, St Louis, Missouri). Gas was sampled via a tubing embedded in the inlet of a VacuMed Universal Ventilation Meter (VacuMed, Ventura, CA, USA), which monitored ventilation frequency (breathing frequency *f*_Br_) and tidal volume (V_T_). Minute ventilation was calculated as V_T_·*f*_Br_. Partial pressures of end-tidal O_2_ (P_ET_O_2_) and CO_2_ (P_ET_CO_2_) were computed as ambient barometric pressure times the expired fractions of O_2_ and CO_2_, respectively. Analog data were continuously digitized online at 250 Hz by a computer interfaced with a data acquisition system (MP150 BIOPAC, Santa Barbara, CA). All measurements were made with the patient wearing a face mask (VacuMed) while resting in the supine position. All tests were performed approximately 2 hours after a light meal, between 9:00 and 11:00 am or 2:00 and 4:00 pm.

### Intermittent Hypoxia Training

**Figure 1.**
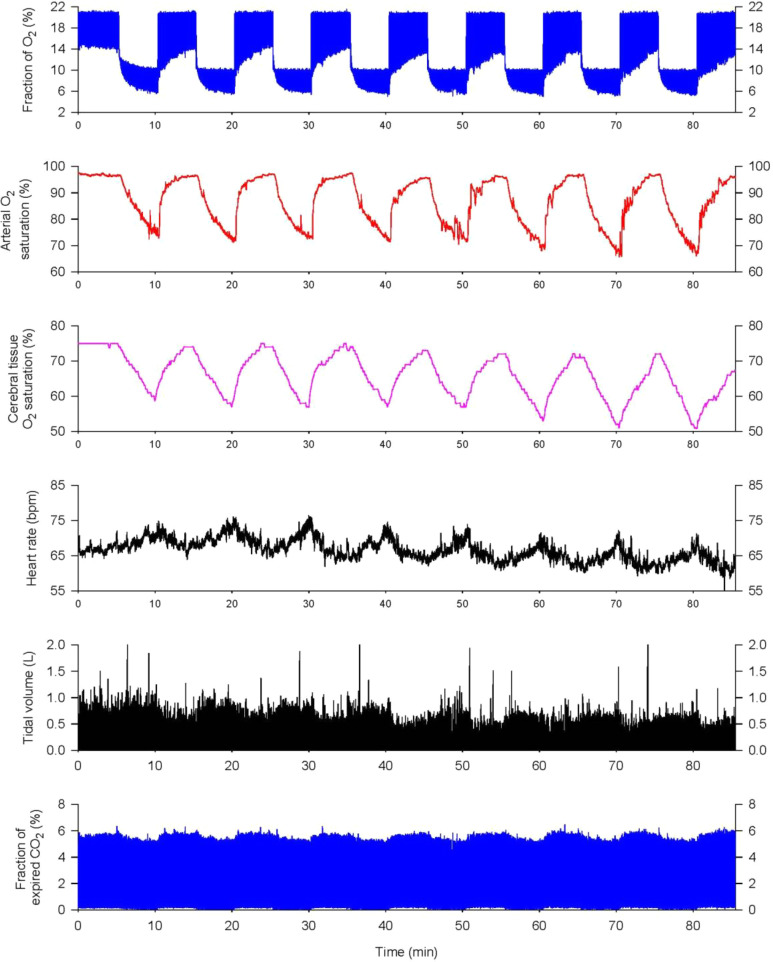
Cardiorespiratory responses to a typical intermittent hypoxia (IH) session. Each IH session consisted of 8 cyclic bouts of breathing 10% O_2_ for 5 minutes and room air for another 5 minutes. From top to bottom, the panels show the fractions of inspired and expired O_2_, arterial O_2_ saturation, cerebral tissue O_2_ saturation, heart rate, tidal volume, and the fraction of expired CO_2_. Arterial O_2_ saturation fell gradually during 5-minute hypoxic exposures and then recovered completely during room-air breathing. Cerebral tissue O_2_ saturation fell in lockstep with arterial O_2_ saturation. Heart rate and tidal volume increased during hypoxia, while end-tidal CO_2_ fell due to increased ventilation.

All participants breathed hypoxic air (10% ± 0.1% O_2_) for 5 min/cycle, alternated with 5-minute room air breathing, repeated for 8 cycles during each IH training (IHT) session (Figure 1), 3 sessions/week for 8 weeks. Two participants’ first 6 training sessions were 10 cycles of 4-minute breathing 10% O_2_ and 4-minute breathing room air, after 2 weeks, these patients were transitioned to the standard 5-minute 10% O_2_ 5-minute room air cycles. This selection was based on the rate of decreases in SaO_2_ and/or ScO_2_ in the orientation IH session. Cumulative hypoxic time was 40 minutes for a single session, and total hypoxia exposure was 960 minutes over the IHT program. Electrocardiogram, SaO_2,_ and ScO_2_ were continuously monitored during all IHT sessions. No adverse or unexpected events were detected in any of the patients during the IHT sessions.

### Data Analysis and Statistics

Each 5-minute hypoxic exposure was divided into five 1-minute intervals, within which SaO_2_, ScO_2,_ HR, SAP, DAP, *f*_Br_, V_T_, minute ventilation, P_ET_CO_2,_ and P_ET_O_2_ values were averaged and compared with the respective prehypoxia baseline values. The reported data represent the average values collected during the last 30 seconds of each 1-minute interval, as described previously.^26^ The V_MCA_ data were collected at rest and during the fifth minute of each hypoxic exposure, respectively, from 5 patients, because 2 patients did not have a clear transcranial Doppler signal. Group data or differences before and after IHT were compared by paired *t* tests after the data set passed the Shapiro-Wilk normality test. Raw cognitive function test scores were reported. Two-factor analyses of variance were applied to assess the effects of the 5-minute hypoxia and IHT. Least-squares linear regression was applied to define the relationship between ScO_2_ and SaO_2_, and a general linear model was applied to evaluate the interactions of IHT with SaO_2_ and ScO_2_ during hypoxia exposures. Statistical analyses were performed with Statistical Analysis System software (SAS Version 9.4, Cary, North Carolina). All data are reported as group mean values ± standard error of the mean. Statistical significance was accepted at P ≤ 0.05.

## Results

### Resting Physiological Variables

Eight-week IHT significantly decreased resting MAP (*P* = .008), SAP (*P* = .047), and DAP (*P* = .031) in older adults with MCI (Table 2). Resting SaO_2_ was slightly (approximately 1%), but significantly (*P* = .037) lower after IHT, possibly due to decreased resting ventilation (*P* = .097). Cerebral tissue oxygenation (ScO_2_) at rest increased (*P* = .035) from 67.9% ± 1.2% before to 70.7% ± 1.6% after IHT (Table 2). However, IHT did not affect (*P* = .207) V_MCA_ at rest (baseline 46.8 ± 3.0 cm/s; post-IHT 44.2 ± 1.9 cm/s). Similarly, CVC at rest was not different (*P* = .632) before (0.479 ± 0.020 cm/mm Hg·s) and after (0.468 ± 0.021 cm/[mm Hg·s]) IHT. Neither V_T_ (*P* = .402), breathing frequency (*f*_Br_, *P* = .117), P_ET_CO_2_ (*P* = .421), nor P_ET_O_2_ (*P* = .554) at rest differed before and after 8-week IHT (Table 2).

**Table 2.** Cardiovascular and Respiratory Function During 5-Minute Acute Hypoxia Before and After IHT.

		0 minute	1 minute	2 minutes	3 minutes	4 minutes	5 minutes	*P* Value
HR, bpm	Before	70 ± 5	72 ± 5	75 ± 5	78 ± 5	80 ± 6	82 ± 5	Hypoxia: .037 IHT: .382
	After	67 ± 5	70 ± 4	73 ± 5	76 ± 4	78 ± 4	78 ± 4	
MAP, mm Hg	Before	101 ± 3	100 ± 3	99 ± 3	98 ± 3	97 ± 3	98 ± 3	Hypoxia: .413 IHT: .021
	After	95 ± 3^a^	97 ± 2	94 ± 2	94 ± 2	92 ± 2	92 ± 2	
SAP, mm H)	Before	139 ± 2	139 ± 2	137 ± 3	136 ± 2	135 ± 3	135 ± 3	Hypoxia: .338 IHT: .006
	After	132 ± 3^a^	135 ± 3	131 ± 4	130 ± 4	128 ± 4	128 ± 4	
DAP, mm Hg	Before	82 ± 3	81 ± 3	80 ± 3	79 ± 3	78 ± 4	79 ± 3	Hypoxia: .319 IHT: .002
	After	77 ± 3^a^	78 ± 2	76 ± 3	76 ± 2	74 ± 2	74 ± 2	
SaO_2,_ %	Before	97.3 ± 0.3	91.6 ± 0.5	83.3 ± 1.4	78.8 ± 1.8	74.8 ± 2.2	70.3 ± 2.9	Hypoxia: .001 IHT: .657
	After	96.3 ± 0.5^a^	90.4 ± 0.7	83.8 ± 0.8	79.7 ± 1.1	76.2 ± 1.5	73.8 ± 1.4	
ScO_2,_ %	Before	67.9 ± 1.2	62.2 ± 1.4	57.7 ± 1.8	55.1 ± 1.9	52.8 ± 2.1	50.9 ± 2.4	Hypoxia: .001 IHT: .004
	After	70.7 ± 1.6^a^	65.9 ± 1.9	61.8 ± 1.7	59.2 ± 1.7	56.7 ± 1.7	55.3 ± 2.0	
*f*_Br,_ br/min	Before	14 ± 1	13 ± 1	13 ± 1	13 ± 1	13 ± 1	13 ± 1	Hypoxia: .872 IHT: .818
	After	12 ± 1	12 ± 2	13 ± 2	13 ± 2	14 ± 2	13 ± 2	
V_T,_ L	Before	0.70 ± 0.06	0.85 ± 0.11	0.95 ± 0.09	0.93 ± 0.09	0.96 ± 0.10	1.08 ± 0.16	Hypoxia: .002 IHT: .597
	After	0.76 ± 0.10	0.93 ± 0.08	0.95 ± 0.09	0.99 ± 0.07	1.00 ± 0.06	1.01 ± 0.11	
V_minute,_ L/min	Before	9.40 ± 0.96	10.46 ± 1.07	11.82 ± 1.31	12.42 ± 1.98	12.24 ± 1.68	13.71 ± 2.21	Hypoxia: .008 IHT: .898
	After	8.66 ± 1.21	11.19 ± 1.47	11.56 ± 1.55	12.58 ± 1.39	13.52 ± 1.70	13.22 ± 2.08	
P_ET_CO_2,_ mm Hg	Before	42.4 ± 0.5	41.0 ± 0.8	40.0 ± 0.6	39.6 ± 1.0	39.8 ± 0.9	39.6 ± 0.9	Hypoxia: .001 IHT: .580
	After	43.1 ± 0.5	41.0 ± 0.3	40.0 ± 0.2	39.3 ± 0.5	38.8 ± 0.7	39.0 ± 0.6	
P_ET_O_2,_ mm Hg	Before	105 ± 2	60 ± 2	49 ± 1	46 ± 1	44 ± 1	42 ± 1	Hypoxia: .001 IHT: .448
	After	103 ± 1	60 ± 1	51 ± 1	48 ± 1	46 ± 1	43 ± 1	

Abbreviations: DAP, diastolic blood pressure; *f*_Br_, breathing frequency; HR, heart rate; IHT, intermittent hypoxia training; MAP, mean arterial pressure; P_ET_CO_2_, partial pressure of end-tidal CO_2_; P_ET_O_2_, partial pressure of end-tidal O_2;_ SAP, systolic blood pressure; SaO_2_, arterial oxygen saturation; ScO_2_, cerebral tissue oxygen saturation; SEM, standard error of the mean; V_T_, tidal volume; Vent, ventilation.

^a^ A statistically significant difference in the baseline valuables (0 minute) before and after IHT (paired *t* test). *P* values indicate the outcome of 2-factor analysis of variance: hypoxia factor (room air vs hypoxic air) and training factor (before vs after IHT). Mean values ± SEM from 7 patients.

### Physiological Responses to Hypoxia Exposures

#### Heart rate, blood pressure, and ventilatory responses to hypoxemia

Five-minute exposures to 10% O_2_ produced moderate hypoxemia (*P* < .001), indicated by progressive decreases in SaO_2_ to approximately 70% and 74% during the fifth minute of hypoxia before and after the 8-week IHT program, respectively. Intermittent hypoxia training did not modulate the SaO_2_ response to acute hypoxia (IHT factor *P* = .657; Table 2). Heart rate progressively increased during 5-minute hypoxia exposures (hypoxia factor *P* = .037), but IHT did not alter these HR responses (IHT factor *P* = .382). Although systemic arterial pressure was not affected by acute hypoxia exposures, MAP, SAP, and DAP were consistently lower after IHT (Table 2). As expected, minute ventilation increased during hypoxia (IHT factor *P* = .008); IHT did not alter this physiological response (IHT factor *P* = .898). The augmented minute ventilation was due entirely to increases in V_T_ (hypoxia factor *P* = .002) at constant *f*_Br_ (hypoxia factor *P* = .872); again, IHT altered neither V_T_ nor *f*_Br_ during hypoxia (Table 2).

#### Cerebral tissue hypoxia and cerebrovascular response during hypoxemia

**Figure 2.**
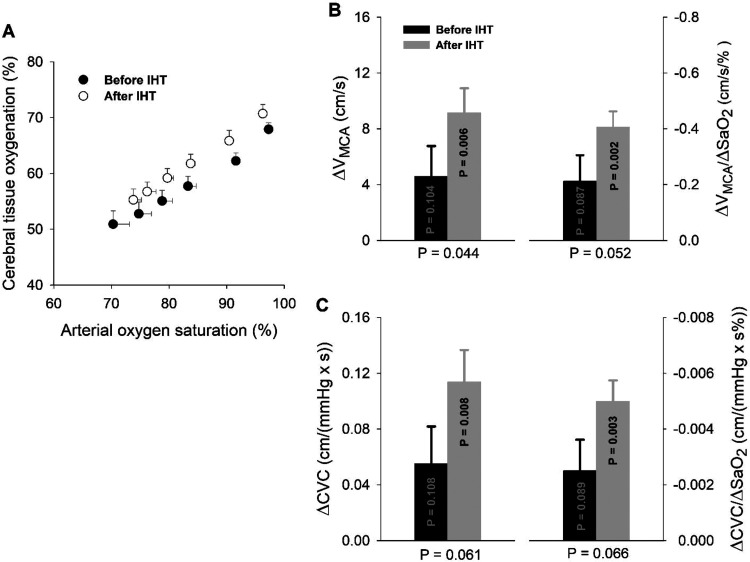
Cerebral tissue oxygenation and cerebrovascular responses during hypoxemia. Panel A, Cerebral tissue hypoxia is highly correlated with hypoxemia (*P* < .001) during 5-minute acute hypoxic exposure, and cerebral tissue O_2_ saturation is consistently greater (*P* = .004) after intermittent hypoxic training (n = 7), but the changes in ScO_2_ per unit change in SaO_2_ are similar (*P* = .232) before and after IHT. Panel B, The changes in blood flow velocity of the middle cerebral artery (ΔV_MCA_) and ΔV_MCA_ values normalized by hypoxemia (ΔV_MCA_/ΔSaO_2_) during the fifth minute of acute hypoxia (n = 5). Although only nonstatistically significant trends in these cerebrovascular responses were noted before IHT (ΔV_MCA_: *P* = .104; ΔV_MCA_/ΔSaO_2_: *P* = .087), IHT’s effects on both were statistically significant (ΔV_MCA_: *P* = .006; ΔV_MCA_/ΔSaO_2_: *P* = .002), and the magnitudes of these ΔV_MCA_ (*P* = .044) and ΔV_MCA_/ΔSaO_2_ (*P* = .052) responses nearly doubled after IHT versus pre-IHT. Panel C, Cerebral vasodilation in terms of changes in estimated cerebrovascular conductance (ΔCVC) or ΔCVC normalized by hypoxemia (ΔCVC/ΔSaO_2_); both measures of vasodilation were appreciably enhanced after 8-week IHT. The *P* values within the bars apply to the changes in these variables during acute hypoxia versus the respective prehypoxia baselines. The *P* values below the abscissa apply to the comparison of IH-induced increases in these variables post- versus pre-IHT (ie, grey bar vs black bar). IHT indicates intermittent hypoxia training; SaO_2,_ arterial oxygen saturation; ScO_2,_ cerebral tissue O_2_ saturation.

During 5-minute hypoxia, ScO_2_ progressively fell (hypoxia factor *P* < .001), concordant with the hypoxia-induced acute hypoxemia (Figure 2A). Although the changes in ScO_2_ per unit change in SaO_2_ were similar before and after IHT, ScO_2_ was consistently greater after IHT versus pre-IHT baseline (*P* = .004). Five-minute hypoxia increased V_MCA_ by 4.5 ± 2.2 cm/s (10% increase) before and 9.2 ± 1.8 cm/s (+21% increase) after IHT, respectively (Figure 2B); thus, IHT doubled the cerebrovascular hyperemic response to acute hypoxia (*P* = .044). This IHT enhancement of cerebrovasodilation remained after normalizing the ΔV_MCA_ values to the decreases in SaO_2_; thus, ΔV_MCA_/ΔSaO_2_ (cm/[s·%]) increased from −0.21 ± 0.09 before to −0.41 ± 0.06 after IHT (*P* = .052). Figure 2C presents the hypoxia-induced changes in estimated CVC (ΔCVC), that is, ΔV_MCA_/MAP, without normalization (left graph) and normalized to the hypoxia-induced decrease in SaO_2_ (ΔCVC/ΔSaO_2_; right graph). Cerebral vasodilation indicated by ΔCVC or ΔCVC/ΔSaO_2_ during the fifth minute of hypoxic breathing was doubled after the IHT program.

### Cognitive Function

Table 3 presents the raw scores of cognitive testing in the participants with aMCI before and after 8-week IHT, except scaled scores are reported for TMT-B because its raw scores failed the normality test. The participants’ performance on MMSE and digit span tests significantly improved after IHT: MMSE score increased from pre-IHT 25.7 ± 0.4 to 27.7 ± 0.6 after IHT (P = 0.038), and digit span score increased from 24.7 ± 1.2 before to 26.1 ± 1.3 after IHT (*P* = .047). Although not statistically significant, a trend toward improvement in the CVLT-II test after versus before IHT (*P* = .102) was noted. However, neither TMT-B nor COWAT was not altered by IHT (Table 3).

**Table 3.** Cognitive Performance Measures Before and After 8-Week Intermittent Hypoxic Training.^a^

	MMSE (Point)		CVLT-II (Total Score)		Digit Span (Total Score)		TMT-B (Scaled Score)^b^		COWAT (Words)	
Subject	Before	After	Before	After	Before	After	Before	After	Before	After
1	24	29	45	40	24	24	5	8	24	26
2	26	30	58	72	23	24	11	14	24	36
3	26	27	66	74	21	21	10	8	28	26
4	25	25	60	60	22	26	8	7	32	19
5	26	27	62	67	29	30	9	9	57	71
6	26	29	42	63	28	31	8	8	33	49
7	27	27	49	51	26	27	1	6	45	48
Mean ± SEM	25.7 ± 0.4	27.7 ± 0.6	54.6 ± 3.5	61.0 ± 4.6	24.7 ± 1.2	26.1 ± 1.3	7.4 ± 1.3	8.6 ± 1.0	34.7 ± 4.6	39.3 ± 6.8
*P* value	.038		.102		.047		.280		.285	

Abbreviations: COWAT, controlled oral word association test; CVLT-II, California verbal learning test-second edition (short version); MMSE, mini-mental state examination; SEM, standard error of the mean; TMT-B, trail making test-version B.

^a^ *P* value indicates the outcome from the paired *t* test.

^b^ Scaled score reported for TMT-B because the unit score (in seconds) failed the normality test for the paired *t* test. All other data represent the raw scores.

## Discussion

Brain has the highest metabolic rate and O_2_ demand of the organs and tissues, and the notion that reduced O_2_ supply could benefit the brain may seem counterintuitive. Indeed, interruptions of O_2_ delivery due to ischemic stroke, cardiac arrest, and asphyxiation inflict devastating damage to the human brain. On the other hand, moderate hypoxia, which causes hypoxemia, but not ischemia, evokes adaptations that increase the brain’s resistance to hypoxia or ischemia.^33^ Moreover, IH-induced hypoxemia activates cerebrovasodilation and may improve cognitive function in the early stages of neurodegenerative disease. This study tested, for the first time, the impact of IHT on cognitive performance and cerebrovascular function in elderly adults with aMCI. Two patients were excluded from the IHT program due to cardiac rhythm disturbances during the orientation screening with acute hypoxia exposure, another 2 could not tolerate the facemask, and another patient did not undergo acute hypoxia due to low SaO_2_ on room air, but of the 9 patients who passed the initial screening, 7 completed the IHT program with no untoward effects, and the other 2 left the study for reasons unrelated to the IHT. Thus, the 8-week program of IH exposures eliciting brief, cyclic, moderate hypoxemia was tolerated by the older adults with aMCI who passed the initial screening without unexpected or advent incidents or cardiac arrhythmias. Furthermore, some measures of cognitive performance, particularly short-term memory and concentration ability, were improved by IHT in these patients with aMCI

The IH exposures lowered resting arterial pressure and enhanced cerebral tissue oxygenation and cerebrovascular dilation. During 5-minute breathing of hypoxia air (10% O_2_), SaO_2_ in older adults with aMCI fell to approximately 70% of baseline before IHT and to approximately 74% of baseline after IHT. This moderate hypoxemia did not cause the elderly participants any distress or discomfort. The cerebral tissue hypoxia induced by moderate hypoxemia was associated with compensatory cerebrovasodilation (Figure 2). Moreover, following 8-week IHT, the patients’ mean, SAP, and DAP were lowered by 5 to 7 mm Hg versus the respective pre-IHT values, bringing them nearer the current targets.^34^ This favorable IHT effect was concordant with those reported by Lyamina et al,^21^ who demonstrated that a 20-day IHT program of 4 to 10 daily cycles of 3-minute exposures to 10% O_2_ alternated with 3-minute room air breathing reduced 24-hour SAP from 151 ± 1 to 129 ± 1 mm Hg and DAP from 95 ± 1 to 79 ± 1 mm Hg in young adult patients (ca 32 years) with stage 1 hypertension. Since 6 of the 7 patients with aMCI had medication-controlled hypertension (Table 1), the present study not only demonstrated the safety of IHT, but extended its antihypertensive effect to older adults with aMCI.

The hemodynamic and ventilatory responses to the hypoxia exposures distinguished the IH exposures from OSA, where interruptions in ventilation produce hypercapnia and provoke hypertension. Intermittent hypoxia exposures augmented the patients’ minute ventilation, an effect entirely due to increased V_T_ (Table 2). Unlike the hypercapnia seen in OSA, the increased ventilation during IH produced acute hypocapnia and, consequently, transient respiratory alkalosis. Moreover, the increased V_T_ would have lowered intrathoracic pressure^35^ and, thus, augmented the cephalothoracic pressure gradient, thereby enhancing cerebral perfusion by facilitating venous return from the brain to the heart.^36^ The augmented cerebral drainage would help maintain cerebral volume homeostasis in the face of compensatory increases in cerebral perfusion during acute hypoxemia. The coordinated augmentation of cerebral perfusion afforded by cerebral vasodilation and V_T_-enhanced cerebrovascular drainage could promote clearance of metabolic wastes and excessive interstitial fluid and solutes from the cerebral parenchyma during cyclic IH exposures.

Although IHT did not modulate resting V_MCA_, the V_MCA_ response to acute hypoxia exposure was appreciably augmented after 8-week IHT. Thus, acute IH exposures provoked cerebral vasodilation and increased cerebral perfusion. It therefore can be concluded that the cerebral tissue hypoxia during IH exposures resulted from hypoxemia, not cerebral ischemia. This repeated cerebrovascular activation over the course of the IHT program may activate beneficial adaptations potentiating hypoxemia-induced cerebral vasodilation in older adults with aMCI.

The IHT program increased resting ScO_2_ in older patients with aMCI. Since IHT did not alter resting V_MCA_, these data suggested that an increased ScO_2_ at rest was more likely associated with an enrichment of microvasculature in the cortical region following IHT. Intermittent hypoxia induction of angiogenesis is likely mediated by increases in vascular endothelial growth factor, erythropoietin, and brain-derived neurotrophic factor after the activated expression of hypoxia-inducible factor 1 (HIF-1) and its target genes.^37^ Erythropoietin, a powerful neuroprotectant^38,39^ which is expressed by astrocytes in a HIF-1 activated manner,^40^ promotes brain angiogenesis after focal ischemia in mice.^41^ In humans, 2-minute moderate IH exposures (reduced P_ET_O_2_ to 45 mm Hg) interspersed with 2-minute recovery increased erythropoietin concentration in blood,^42^ enabling sufficient circulating erythropoietin to cross the blood–brain barrier to afford neuroprotection.^43^ Furthermore, hypoxia adaptation is reported to augment cerebral expression of neuroglobin, an O_2_-binding heme protein, in rat,^44^ particularly in the ischemia-vulnerable hippocampus.^45^ Augmented neuroglobin combined with angiogenesis may provide the basis for the IHT-related increase in ScO_2_ observed in the present study.

In parallel with enhanced cerebral tissue oxygenation, this study also demonstrated that IHT improved the participants’ MMSE and digit span scores, and tended to improve their performance on the CVLT-II test (Table 3). Although TMT-B and COWAT performance improved in 4 and 5 of the 7 participants, respectively, neither of these improvements attained statistical significance following the 8-week IHT program. This discrepancy was probably associated with an age-related increase in intraindividual variability in executive function and/or reaction time^46-48^ as indicated by TMT-B and COWAT scores, in addition to a possible type II or β error. Nonetheless, the improved short-term memory and concentration ability following IHT suggested that this intervention could be potentially applied to help improve overall cognitive function in patients with aMCI. The persistence of the IHT-induced cognitive improvements and the possibility that more protracted IHT programs may produce even greater benefits, merit investigation.

### Study Limitations and Perspectives

The main study limitation was that we were unable to distinguish whether improvement in some cognitive performance tests resulted from the IHT intervention or a practice effect due to the study’s repeated-measures design, because there was no sham-IHT or control group in this pilot study. In addition, the patients might experience a placebo effect since the intervention was not blind. Future clinical trials utilizing a randomized, double-blind design to compare pre- and postintervention cognitive performance in IHT versus sham-IHT groups are needed. Nonetheless, this pilot study suggests that IHT with brief cyclic moderate hypoxemia is tolerated in elderly with aMCI and potentially effective to prevent or retard neurodegenerative progression to AD-dementia before cognitive decline becomes irreversible. To assure patient safety, before IHT commences, the patient should undergo acute hypoxia exposure while electrocardiogram, SaO_2,_ and ScO_2_ are monitored, as was done in the orientation visit of this pilot study.

In summary, this exploratory pilot study confirms that brief, cyclic moderate hypoxemia is tolerable by elderly adults. Intermittent hypoxia training may be adapted as a novel intervention for treating patients with MCI or early AD. An 8-week IHT program lowers resting arterial pressure and enhances cerebrocortical tissue O_2_ saturation and cerebral vasodilation during hypoxia challenge and potentially improves cognitive performance on tests for attention and short-term memory in older adults with MCI.

## References

[1] Serebrovskaya TV Manukhina EB Smith ML Downey HF Mallet RT . Intermittent hypoxia: cause of or therapy for systemic hypertension? Exp Biol Med (Maywood). 2008;233(6):627–650. 18408145 10.3181/0710-MR-267

[2] Navarrete-Opazo A Mitchell GS . Therapeutic potential of intermittent hypoxia: a matter of dose. Am J Physiol Regul Integr Comp Physiol. 2014;307(10):R1181–1197. 25231353 10.1152/ajpregu.00208.2014 PMC4315448

[3] Tahawi Z Orolinova N Joshua IG Bader M Fletcher EC . Altered vascular reactivity in arterioles of chronic intermittent hypoxic rats. J Appl Physiol. 2001;90(5):2007–2013; discussion 2000. 11299297 10.1152/jappl.2001.90.5.2007

[bibr4-1533317519896725] Phillips SA Olson EB Morgan BJ Lombard JH . Chronic intermittent hypoxia impairs endothelium-dependent dilation in rat cerebral and skeletal muscle resistance arteries. Am J Physiol Heart Circ Physiol. 2004;286(1):H388–393. 14512283 10.1152/ajpheart.00683.2003

[5] Wu Q Cunningham JT Mifflin S . Transcription factor ΔFosB acts within the nucleus of the solitary tract to increase mean arterial pressure during exposures to intermittent hypoxia. Am J Physiol Heart Circ Physiol. 2018;314(2):H270–H277. 29101166 10.1152/ajpheart.00268.2017 PMC5867648

[6] Xu XM Yao D Cai XD , et al. Effect of chronic continual- and intermittent hypoxia-induced systemic inflammation on the cardiovascular system in rats. Sleep Breath. 2015;19(2):677–684. 25395264 10.1007/s11325-014-1075-9

[7] Li RC Row BW Kheirandish L , et al. Nitric oxide synthase and intermittent hypoxia-induced spatial learning deficits in the rat. Neurobiol Dis. 2004;17(1):44–53. 15350964 10.1016/j.nbd.2004.05.006

[8] Gozal D Nair D Goldbart AD . Physical activity attenuates intermittent hypoxia-induced spatial learning deficits and oxidative stress. Am J Respir Crit Care Med. 2010;182(1):104–112. 20224062 10.1164/rccm.201001-0108OC PMC2902754

[9] Ramond A Ribuot C Levy P Joyeux-Faure M . Deleterious myocardial consequences induced by intermittent hypoxia are reversed by erythropoietin. Respir Physiol Neurobiol. 2007;156(3):362–369. 17161979 10.1016/j.resp.2006.10.011

[10] Wang Y Guo SZ Bonen A , et al. Monocarboxylate transporter 2 and stroke severity in a rodent model of sleep apnea. J Neurosci. 2011;31(28):10241–10248. 21753001 10.1523/JNEUROSCI.1462-11.2011 PMC3164586

[11] Mallet RT Ryou MG Williams AG Jr Howard L Downey HF . β_1_-Adrenergic receptor antagonism abrogates cardioprotective effects of intermittent hypoxia. Basic Res Cardiol. 2006;101(5):436–446. 16705468 10.1007/s00395-006-0599-y

[bibr12-1533317519896725] Manukhina EB Belkina LM Terekhina OL , et al. Normobaric, intermittent hypoxia conditioning is cardio- and vasoprotective in rats. Exp Biol Med (Maywood). 2013;238(12):1413–1420. 24189016 10.1177/1535370213508718

[13] Zong P Setty S Sun W , et al. Intermittent hypoxic training protects canine myocardium from infarction. Exp Biol Med (Maywood). 2004;229(8):806–812. 15337835 10.1177/153537020422900813

[14] Zhu XH Yan HC Zhang J , et al. Intermittent hypoxia promotes hippocampal neurogenesis and produces antidepressant-like effects in adult rats. J Neurosci. 2010;30(38):12653–12663. 20861371 10.1523/JNEUROSCI.6414-09.2010 PMC6633584

[bibr15-1533317519896725] Dale EA Ben Mabrouk F Mitchell GS . Unexpected benefits of intermittent hypoxia: enhanced respiratory and nonrespiratory motor function. Physiology (Bethesda). 2014;29(1):39–48. 24382870 10.1152/physiol.00012.2013 PMC4073945

[bibr16-1533317519896725] Goryacheva AV Kruglov SV Pshennikova MG , et al. Adaptation to intermittent hypoxia restricts nitric oxide overproduction and prevents beta-amyloid toxicity in rat brain. Nitric Oxide. 2010;23(4):289–299. 20804853 10.1016/j.niox.2010.08.005

[bibr17-1533317519896725] Jung ME Simpkins JW Wilson AM Downey HF Mallet RT . Intermittent hypoxia conditioning prevents behavioral deficit and brain oxidative stress in ethanol-withdrawn rats. J Appl Physiol. 2008;105(2):510–517. 18499779 10.1152/japplphysiol.90317.2008 PMC2519950

[18] Ryou MG Mallet RT Metzger DB Jung ME . Intermittent hypoxia training blunts cerebrocortical presenilin 1 overexpression and amyloid-beta accumulation in ethanol-withdrawn rats. Am J Physiol Regul Integr Comp Physiol. 2017;313(1): R10–R18. 28490448 10.1152/ajpregu.00050.2017 PMC5538853

[19] Burtscher M Pachinger O Ehrenbourg I , et al. Intermittent hypoxia increases exercise tolerance in elderly men with and without coronary artery disease. Int J Cardiol. 2004;96(2):247–254. 15262041 10.1016/j.ijcard.2003.07.021

[20] Haider T Casucci G Linser T , et al. Interval hypoxic training improves autonomic cardiovascular and respiratory control in patients with mild chronic obstructive pulmonary disease. J Hypertens. 2009;27(8):1648–1654. 19387363 10.1097/HJH.0b013e32832c0018

[21] Lyamina NP Lyamina SV Senchiknin VN Mallet RT Downey HF Manukhina EB . Normobaric hypoxia conditioning reduces blood pressure and normalizes nitric oxide synthesis in patients with arterial hypertension. J Hypertens. 2011;29(11):2265–2272. 21897291 10.1097/HJH.0b013e32834b5846

[22] Saeed O Bhatia V Formica P , et al. Improved exercise performance and skeletal muscle strength after simulated altitude exposure: a novel approach for patients with chronic heart failure. J Card Fail. 2012;18(5):387–391. 22555269 10.1016/j.cardfail.2012.02.003

[23] Hayes HB Jayaraman A Herrmann M Mitchell GS Rymer WZ Trumbower RD . Daily intermittent hypoxia enhances walking after chronic spinal cord injury: a randomized trial. Neurology. 2014;82(2):104–113. 24285617 10.1212/01.WNL.0000437416.34298.43 PMC3897437

[24] Serebrovska TV Portnychenko AG Drevytska TI , et al. Intermittent hypoxia training in prediabetes patients: Beneficial effects on glucose homeostasis, hypoxia tolerance and gene expression. Exp Biol Med (Maywood). 2017;242(15):1542–1552. 28758418 10.1177/1535370217723578 PMC5648288

[25] Bayer U Likar R Pinter G , et al. Intermittent hypoxic-hyperoxic training on cognitive performance in geriatric patients. Alzheimers Dement (NY). 2017;3(1):114–122. 10.1016/j.trci.2017.01.002 PMC5651371 29067323

[26] Zhang P Downey HF Shi X . Acute intermittent hypoxia exposures enhance arterial oxygen delivery. Exp Biol Med (Maywood). 2010;235(9):1134–1141. 20660087 10.1258/ebm.2010.009393

[27] Liu X Xu D Hall JR , et al. Enhanced cerebral perfusion during brief exposures to cyclic intermittent hypoxemia. J Appl Physiol. 2017;123(6):1689–1697. 29074711 10.1152/japplphysiol.00647.2017

[28] Petersen RC Smith GE Waring SC Ivnik RJ Kokmen E Tangelos EG . Aging, memory, and mild cognitive impairment. Internat Psychoger. 1997;9(suppl 1):65–69. 10.1017/s1041610297004717 9447429

[29] Zhang P Huang G Shi X . Cerebral vasoreactivity during hypercapnia is reset by augmented sympathetic influence. J Appl Physiol. 2011;110(2):352–358. 21071587 10.1152/japplphysiol.00802.2010 PMC3043785

[bibr30-1533317519896725] Formes K Zhang P Tierney N Schaller F Shi X . Chronic physical activity mitigates cerebral hypoperfusion during central hypovolemia in elderly humans. Am J Physiol Heart Circ Physiol. 2010;298(3): H1029–1037. 20044443 10.1152/ajpheart.00662.2009 PMC2838545

[bibr31-1533317519896725] Guo H Tierney N Schaller F Raven PB Smith SA Shi X . Cerebral autoregulation is preserved during orthostatic stress superimposed with systemic hypotension. J Appl Physiol. 2006;100(6):1785–1792. 16424075 10.1152/japplphysiol.00690.2005

[32] Zhang P Shi X Downey HF . Two-week normobaric intermittent-hypoxic exposures stabilize cerebral vasoreactivity during hypocapnia and hypercapnia. Exp Biol Med (Maywood). 2015;240(7):961–968. 25504012 10.1177/1535370214562339 PMC4935400

[33] Baillieul S Chacaroun S Doutreleau S Detante O Pepin JL Verges S. Hypoxic conditioning and the central nervous system: a new therapeutic opportunity for brain and spinal cord injuries? Exp Biol Med (Maywood). 2017;242(11):1198–1206. 28585890 10.1177/1535370217712691 PMC5478009

[34] Whelton PK Carey RM Aronow WS , et al. 2017 ACC/AHA/AAPA/ABC/ACPM/AGS/APhA/ASH/ASPC/NMA/PCNA guideline for the prevention, detection, evaluation, and management of high blood pressure in adults: executive summary: a report of the American college of cardiology/American heart association task force on clinical practice guidelines. Circulation. 2018;138(17): e426–e483. 30354655 10.1161/CIR.0000000000000597

[35] Moreno AH Burchell AR Van der Woude R Burke JH . Respiratory regulation of splanchnic and systemic venous return. Am J Physiol. 1967;213(2):455–465. 6036333 10.1152/ajplegacy.1967.213.2.455

[36] Convertino VA Ryan KL Rickards CA , et al. Optimizing the respiratory pump: harnessing inspiratory resistance to treat systemic hypotension. Respir Care. 2011;56(6):846–857. 21333089 10.4187/respcare.01018 PMC6607101

[37] Bernaudin M Nedelec AS Divoux D MacKenzie ET Petit E Schumann-Bard P . Normobaric hypoxia induces tolerance to focal permanent cerebral ischemia in association with an increased expression of hypoxia-inducible factor-1 and its target genes, erythropoietin and VEGF, in the adult mouse brain. J Cereb Blood Flow Metab. 2002;22(4):393–403. 11919510 10.1097/00004647-200204000-00003

[38] Mallet RT Ryou M . Erythropoietin: endogenous protection of ischemic brain. Vitam Horm: 2017;105:197–232. 28629519 10.1016/bs.vh.2017.01.002

[39] Rabie T Marti HH . Brain protection by erythropoietin: a manifold task. Physiology (Bethesda). 2008;23(5):263–274. 18927202 10.1152/physiol.00016.2008

[40] Ruscher K Freyer D Karsch M , et al. Erythropoietin is a paracrine mediator of ischemic tolerance in the brain: evidence from an in vitro model. J Neurosci. 2002;22(23):10291–10301. 12451129 10.1523/JNEUROSCI.22-23-10291.2002 PMC6758760

[41] Li Y Lu Z Keogh CL Yu SP Wei L . Erythropoietin-induced neurovascular protection, angiogenesis, and cerebral blood flow restoration after focal ischemia in mice. J Cereb Blood Flow Metab. 2007;27(5):1043–1054. 17077815 10.1038/sj.jcbfm.9600417

[42] Brugniaux JV Pialoux V Foster GE , et al. Effects of intermittent hypoxia on erythropoietin, soluble erythropoietin receptor and ventilation in humans. Eur Resp J. 2011;37(4):880–887. 10.1183/09031936.00156009 20947680

[43] Brines ML Ghezzi P Keenan S , et al. Erythropoietin crosses the blood-brain barrier to protect against experimental brain injury. Proc Natl Acad Sci USA. 2000;97(19):10526–10531. 10984541 10.1073/pnas.97.19.10526 PMC27058

[44] Avivi A Gerlach F Joel A , et al. Neuroglobin, cytoglobin, and myoglobin contribute to hypoxia adaptation of the subterranean mole rat Spalax. Proc Natl Acad Sci USA. 2010;107(50):21570–21575. 21115824 10.1073/pnas.1015379107 PMC3003035

[45] Wu Q Yu KX Ma QS Liu YN . Effects of intermittent hypobaric hypoxia preconditioning on the expression of neuroglobin and Bcl-2 in the rat hippocampal CA1 area following ischemia-reperfusion. Genet Mol Res. 2015;14(3):10799–10807. 26400308 10.4238/2015.September.9.18

[46] Hultsch DF MacDonald SW Dixon RA . Variability in reaction time performance of younger and older adults. J Gerontol B Psychol Sci Soc Sci. 2002;57(2): P101–115. 11867658 10.1093/geronb/57.2.p101

[bibr47-1533317519896725] West R Murphy KJ Armilio ML Craik FI Stuss DT . Lapses of intention and performance variability reveal age-related increases in fluctuations of executive control. Brain Cogn. 2002;49(3):402–419. 12139961 10.1006/brcg.2001.1507

[48] Dykiert D Der G Starr JM Deary IJ . Age differences in intra-individual variability in simple and choice reaction time: systematic review and meta-analysis. PLoS One. 2012;7(10):e45759. 23071524 10.1371/journal.pone.0045759 PMC3469552

